# Management effect on bird and arthropod interaction in suburban woodlands

**DOI:** 10.1186/1472-6785-11-8

**Published:** 2011-03-01

**Authors:** Erik Heyman, Bengt Gunnarsson

**Affiliations:** 1 Department of Plant and Environmental Sciences, University of Gothenburg, Box 461, SE-405 30 Göteborg, Sweden

## Abstract

**Background:**

Experiments from a range of ecosystems have shown that insectivorous birds are important in controlling the populations of their invertebrate prey. Here, we report on a large field experiment testing the hypothesis that management for enhancing recreational values in suburban woodlands affects the intensity of bird predation on canopy-living arthropods. Bird exclosures were used in two types of management (understory clearance and dense understory) at two foraging heights in oak *Quercus robur *canopies and the experiment was replicated at two sites.

**Results:**

The biomass and abundance of arthropods were high on net-enclosed branches but strongly reduced on control branches in both types of management. In woods with dense understory, the effect of bird predation on arthropod abundance was about twice as high as in woods with understory clearance. The effect of bird predation on arthropod biomass was not significantly affected by management.

**Conclusions:**

Our data provide experimental evidence to support the idea that bird predation on arthropods can be affected by forest management. We suggest that the mechanism is twofold: reduction of bird abundance and shift of foraging behaviour. In urban woodlands, there may be a management trade-off between enhancing recreational values and promoting bird predation rates on arthropods.

## Background

Avian insectivores are known to reduce pest populations and this ecosystem service may be of high value to, e.g., agriculture and forestry [1-4]. However, this important function is at risk in certain habitats [2,3,5,6]. For instance, bird populations in farmlands and forests have been declining for a long time in Europe [7-10]. Urbanization causes both habitat loss and fragmentation of nature remnants and there are numerous reports of negative effects on bird diversity and abundance due to habitat change and fragmentation in urban areas [11-14]. Christie and Hochuli [15] found elevated levels of leaf damage caused by herbivorous insects in highly fragmented urban forests, which were hypothesized to be caused by reduced populations of insectivorous birds.

Studies in many contrasting forest systems suggest that birds exert a significant impact on arboreal arthropod populations [16-21]. Recent meta-analyses confirm that insectivorous birds strongly reduce abundances of arthropod herbivores, as well as arthropod predators, and thereby enhance plant performance [22,23]. Specifically, insectivores contribute significantly to controlling pest populations in agroforestry systems and a number of variables, such as vegetation strata, habitat structure and bird abundance and diversity, affect the intensity of bird predation pressure on arthropods [22]. This draws attention to the importance of human impact, and the role of management is paramount in coping with habitat change and simultaneously enhancing ecosystem services such as biological control [24,25].

Earlier experimental studies in forest ecosystems in southern Sweden have shown that bird predation effects on canopy-living arthropods are generally strong. Avian insectivores reduce arthropod abundances in winter [16,26], as well as in summer [27,28], and reduce the mean size of arthropods in both managed coniferous forests [27] and deciduous stands in city parks and suburban woodlands [28]. Taken together these studies provide substantial evidence that avian predators contribute to controlling arthropod abundances in forests of southern Sweden. We therefore anticipated that birds in our experiment would affect their arthropod prey in a similar way.

In a large-scale, replicated field experiment, we examined effects of forest management on bird predation pressure on arthropods. The form of management studied was clearance of understory in suburban, oak-dominated woodlands. Clearance of understory is a frequently used type of management in urban and suburban woodlands in Sweden [29] as relatively open forests are generally favoured by the public [30,31]. Removal of bushes and shrubs may have an impact on the abundance and behaviour of birds and if so, it might indirectly affect the intensity of predation. At the same sites as in the present study, a parallel investigation of management effects on breeding bird density and diversity was conducted. In 2008, two years after management, breeding bird densities had decreased by on average 37% in the cleared plots compared to unmanaged plots [32].

Our main hypothesis was that management would affect the predator-prey relationship by altering the intensity of bird predation on canopy-living arthropods. We conducted an exclosure experiment to first examine whether arthropods were reduced by avian insectivores. Secondly, the intensity of predation pressure was measured by differentials of arthropod abundance between canopies with and without bird exclosures. We then examined the influence of management, foraging height and site on bird predation pressure.

## Results

**Figure 1:**
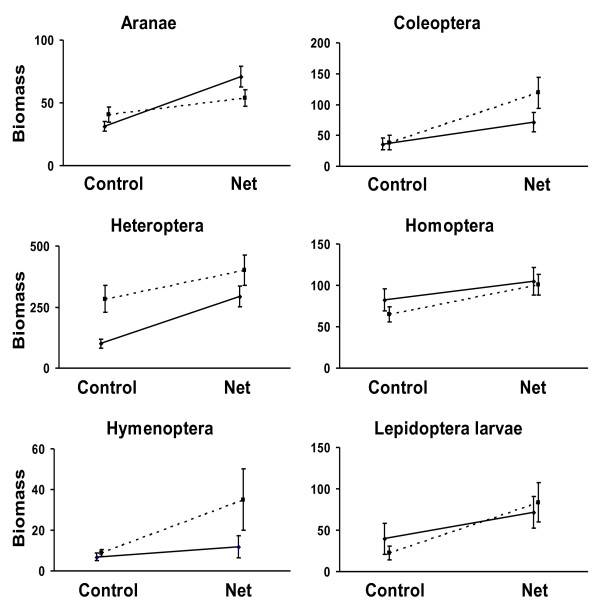
**Arthropod biomass**. Arthropod biomass (g wet arthropod biomass/kg wet leaf mass ± S.E.) of the six largest orders in relation to experimental treatment and management (dotted line = dense understory, full line = understory clearance).

Effects of the bird exclosures across sites, management types and height levels were found to be strong for both arthropod abundance (t = -3.441, df = 87, p = 0.001) and biomass (t = -7.356, df = 87, p < 0.001). Arthropod biomass was more strongly affected than arthropod abundance by the net exclosures. On average, arthropod biomass was twice as high on net-enclosed branches (0.81 ± 0.05 mg arthropods/g wet leaf mass, mean ± S.E., n = 88) than on control branches (0.42 ± 0.04, n = 88). Arthropod biomass of the six largest orders in relation to experimental treatment and management is shown in Figure 1. Arthropod abundance was on average 20% higher on net-enclosed branches (0.30 ± 0.02 individuals/g wet leaf mass) than on control branches (0.25 ± 0.02). The abundances and biomass of various arthropod orders in relation to experimental treatment and management are shown in Additional file 1.

The relative difference between net-enclosed and control branches (predation effect, *E*, see Methods) was tested in relation to Management, Height and Site and the first-order interactions between these factors. The predation effect was not correlated for high and low pairs of branches, either for biomass (r_s _= 0.067, p = 0.536) or for abundance (r_s _= 0.191, p = 0.074). Therefore, each pair of branches (control and net-enclosed) was treated as an independent sample.

**Figure 2:**
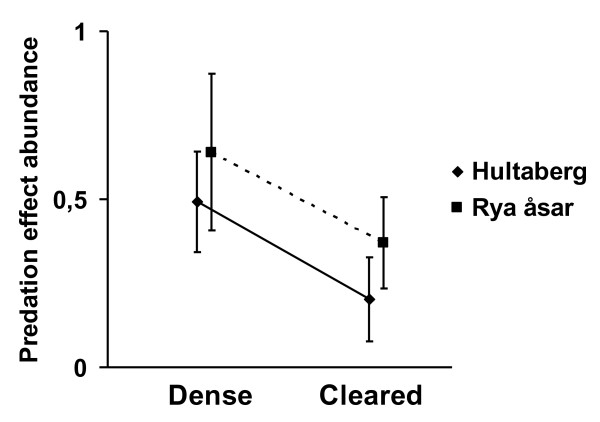
**Bird predation effect on arthropod abundance**. Bird predation effect (± S.E.) on arthropod abundance in relation to management (dense understory and understory clearance) and site (full line = Hultaberg, dotted line = Rya åsar).

**Figure 3:**
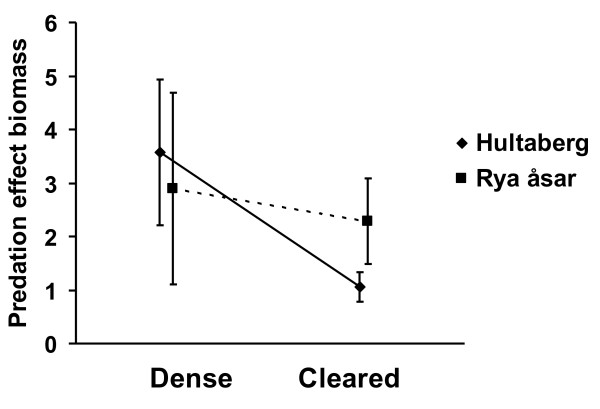
**Bird predation effect on arthropod biomass**. Bird predation effect (± S.E.) on arthropod biomass in relation to management (dense understory and understory clearance) and site (full line = Hultaberg, dotted line = Rya åsar).

Predation effects on arthropod abundance differed significantly by a factor of about two due to management (Table 1, p = 0.022). The predation effect on arthropod abundance was 0.56 ± 0.13 (mean ± S.E.) in the control areas and 0.28 ± 0.09 in the cleared areas (Figure 2). The predation effect was almost six times larger on arthropod biomass than on arthropod abundance, although the predation effect on arthropod biomass was not significantly affected by management (Table 1, p = 0.559). Predation effect on arthropod biomass was 3.27 ± 1.09 in the control areas and 1.62 ± 0.40 in the managed areas (Figure 3).

**Table 1:** ANOVA output

	Predation effect biomass				Predation effect abundance			
	**df**	**MS**	**F**	**p**	**df**	**MS**	**F**	**p**

Management	1, 1	0.138	0.691	0.559	1, 1	1.713	807.253	0.022

Height	1, 1	0.013	2.787	0.344	1, 1	0.098	0.069	0.837

Site	1, 0.056	0.042	0.889	0.891	1, 0.056	0.539	0.632	0.702

Management × Height	1, 81	0.000	0.001	0.978	1, 81	0.000	0.001	0.979

Management × Site	1, 81	0.200	1.272	0.263	1, 81	0.002	0.004	0.952

Height × Site	1, 81	0.005	0.030	0.863	1, 81	1.431	2.467	0.120

ANOVA on predation effect of biomass (mg/g wet leaf-mass, log (x + 1) transformed) and abundance (numbers/g wet leaf-mass) in relation to management (understory clearance and dense understory), foraging height (low and high branches) and site (Hultaberg and Rya åsar).

No significant height or site effects were found (Table 1), although the mean values of the predation effect were lower on high branches (12-15 m above the ground) than on low branches (3-5 m above the ground) for both arthropod abundance and biomass. None of the tested interactions (Management × Height, Management × Site, Height × Site) were found to be statistically significant for either arthropod abundance or biomass.

## Discussion

Our results support previous studies that have shown that insectivorous birds significantly decrease arthropod populations in forest and agricultural ecosystems [6,22,23,33]. Even though our exclosure experiment was a relatively short-term study (4 weeks), the effects of bird predation on arthropod abundance were about twice as large in control areas as in managed areas. The mean difference between management types regarding the predation rate on arthropod biomass was similar to that on arthropod abundance, although it was not statistically significant in either case. There were large variations among samples in the biomass data, probably due to a few large arthropod individuals such as Lepidoptera larvae or Heteroptera that were present on some of the branches. Our data suggest that the potential for population control of arthropods is higher in the areas with dense understory than in the cleared areas. Interestingly, our result deviates partly from the conclusions in the meta-analysis by Philpott *et al*. [33] of bird predation effects in tropical agroforests. They found that differences in vegetation complexity in coffee and cacao agroforests (including tree richness, tree density and canopy cover) did not explain the rate of arthropod removal by insectivorous birds. A possible explanation for our deviating results is that the oak forests in our study are structurally more complex, with denser and more variable understory vegetation, compared to the agroforest habitats included in the meta-analysis.

Contrary to our hypothesis, no height effects or height-management interactions on bird predation effect were found. The removal of the understory did not increase bird predation in the lower oak canopies in the managed plots, which might have been expected if birds that predominantly forage in the understory had remained at the same sites after clearance. An exclosure experiment in bush canopies, conducted at the same location as in the present study, showed strong bird predation effects on arthropods in bush canopies, which indicates that prey found in the understory is an important food resource in these forests [34].

Theory suggests that top-down effects of predation will be stronger where complexity and diversity are lower [22,35]. Based on theoretical predictions, we could have expected stronger impacts of bird predation in the cleared areas than in the areas with dense understory because as bushes and small trees were removed, the forest became structurally less complex. In contrast to these theories, we found that bird predation pressure in managed areas was about half the predation pressure of that in control plots. We suggest that the mechanism of changing avian predation is twofold. Firstly, the bird abundance decreased on managed plots. As mentioned earlier, a parallel investigation of management effects on breeding bird density and diversity was conducted before and after the clearance of understory. Territory mapping showed that densities of insectivorous birds decreased by on average 37% in the cleared plots compared to unmanaged plots while bird diversity was not affected by the management [32]. Secondly, reduced pressure on arthropods can be a result of a shift in bird foraging behaviour. Possibly, birds avoid foraging in the more open managed plots to reduce the risk of exposure to predators such as sparrowhawk *Accipiter nisus *[36]. These two mechanisms may act simultaneously, decreasing options for avian control of arthropod abundance.

We did not quantify the human presence at the experimental sites. Trail densities are high at both sites and the forests are frequently used by the public for walking, jogging and other recreational activities (personal observation). As the understory was cleared there may have been more human movement in the cleared areas due to higher recreational qualities. This may have increased bird disturbance and acted as an additional mechanism of lower foraging rates in the cleared areas.

Several earlier studies have shown that bird predation can contribute to dampening the outbreaks of forest pests, even though such predation can only be effective at controlling invertebrate populations at low to moderate densities [3,4,37]. In our study, we did not conduct separate analyses of bird predation rates on predaceous and herbivorous arthropods. This was mainly due to small sample sizes of the separate arthropod orders. Some of the largest orders in our study, e.g. Coleoptera, Heteroptera and Hymenoptera also include both predaceous and herbivorous species. The evaluation of management effects on ecosystem services, such as a reduction of leaf damage due to bird predation on herbivorous arthropods is, therefore, complicated. However, a recently published meta-analysis showed that, as opposed to theoretical predictions, predators feeding on both herbivores and predators, or intraguild predation, strengthened, rather than weakened, trophic cascades such as reduction of plant damage [23]. Several other studies have shown that bird predation on arthropods can cause reduced leaf damage and increase plant biomass [38,39]. Further studies are needed to confirm whether the lower bird predation rate on the total abundance of tree-living arthropods that we found in managed areas could cause increased leaf damage.

Experiments from a range of ecosystems have shown that insectivorous birds are important in controlling the populations of their invertebrate prey [1,40-43]. There are, however, few experimental tests on the effects of habitat management on bird predation effects. A recent meta-analysis by Van Bael *et al*. [22] concluded that higher bird richness is associated with greater arthropod removal but no difference in the magnitude of bird effects was observed between systems with different habitat structure and plant diversity. However, two recently published comparative studies [5,44] suggested that vegetation structure could influence predator control of pest populations. The results from our study showed differences in bird predation due to variation in vegetation structure (i.e. management) even though bird diversity was not affected. More experimental work in tropical, temperate and boreal systems is needed to evaluate possible differences in mechanisms of the interaction between birds and their arthropod prey. Further experimental studies are also needed to elucidate the importance of management to pest control by insectivorous birds.

Our experiments were conducted at recreational sites in suburban woodlands with deciduous forest. Swedish forests are generally dominated by coniferous trees (on average 85%) but deciduous trees comprise about 50% of the urban and suburban woodlands in Sweden [29]. These woodlands are mainly owned by municipalities and are to a large extent managed to promote social and recreational values [45,46]. There are high social values associated with clearance of understory as the general public seems to prefer relatively open forests with a low density of shrubs and bushes [30]. Birds also contribute to the social values of the forests as they have been shown to be the most highly valued animals in urban green areas and the experience of seeing wild birds is recognized as an important cultural ecosystem service [4,47]. Our management experiment showed that both bird abundance [35] and the effect of bird predation on canopy-living arthropods decreased after extensive clearance of understory.

## Conclusions

Our data provide experimental evidence to support the idea that bird predation pressure on arthropods can be affected by forest management. The field experiment showed that the effects of bird predation on arthropod abundance were larger in areas with dense understory than in areas with understory clearance. The reduction of bird predation on arboreal arthropods in the managed areas may also have negative effects on recreational values if relaxed control of arthropods increases leaf damage on trees. Although exclosure experiments are widely used to quantify bird predation on arthropods, our study is, to our knowledge, the first to show experimentally that forest management can affect naturally occurring predation pressure on arthropod abundance. We conclude that there is a possible conflict of interests in the management of urban woodlands. Management for enhanced aesthetic values of the forest, such as clearance of understory, may have a negative impact on the biological control of tree-living arthropods. Partial clearance of understory near paths and frequently visited areas is suggested as a preferable management strategy compared to complete removal of understory in suburban woodlands. This would promote recreational values and probably minimize the negative impact on forest bird abundance and foraging rates on arthropods.

## Methods

### Study sites and management

The study was conducted in the province of Västra Götaland, southwest Sweden. Biogeographically this region includes the vegetational border separating the southern deciduous forest region (nemoral) and the southern coniferous forest region (boreo-nemoral).The experiment was conducted at two sites, Hultaberg and Rya åsar, both located in woodlands on the outskirts of the city of Borås (63 000 inhabitants, 57°43'N,12°56'E).

The Hultaberg site (area 8.0 ha) is located south of the town, surrounded by oak forests on two sides and a residential district and an industrial estate on the other two sides. The dominating tree species at Hultaberg were Oak *Quercus robur *(87% of the stems >30 cm dbh), followed by Birch *Betula spp*. (9%), and Scots pine *Pinus sylvestris *(4%). The understory consisted mainly of Rowan *Sorbus aucuparia *(52% of the stems 0-10 cm dbh), Alder buckthorn *Frangula alnus *(31%), Birch *Betula spp*. (6%) and Oak *Quercus robur *(5%).

The other site, Rya åsar (area 7.2 ha), is part of a municipal nature reserve in a large forest (550 ha) located on the northern outskirts of the town. The site is surrounded by a road on one side, coniferous forest on one side and oak forests on the remaining two sides. The most common large trees were *Q. robur *(78% of the stems >30 cm dbh), *Betula spp*.(18%) and *S. aucuparia *(2%). Understory (0-10 cm dbh) species were mainly *S. aucuparia *(44%), *F. alnus *(30%), *Betula spp*. (7%) and Hazel *Corylus avellana *(7%).

The management experiment was initiated at the study sites in 2006. Understory was cleared in order to evaluate management effects on birds, arthropods and forest recreational values. None of the sites had been subject to any recent (<10 years) clearance or thinning before the experiment. The understory was therefore well developed with dense vegetation, consisting of bushes, shrubs and low trees. Results regarding management effects on bird abundance and diversity were reported in Heyman [32]. Understory clearance was conducted at both study sites in an area of 4.0 and 3.9 hectares (Hultaberg and Rya åsar, respectively) and there were control areas of about equal size (4.0 and 3.3 hectares) at both sites. The management treatment was randomly assigned to each area. In the cleared areas, about 90 percent of the bushes, shrubs and small trees with a base diameter of less than 10 cm were removed while the dense understory areas were left unmanaged and served as controls. Densities of bushes and small trees before and after management are shown in Table 2. Clearance of understory was carried out by municipal forestry workers during autumn 2006 and early winter 2007 and the woody debris from the clearance was transported out of the forest.

**Table 2:** Density of bushes and small trees

Site	Management	Before	After
Hultaberg	Cleared	143.8 (15.5)	17.9 (2.4)

Hultaberg	Dense understory	84.2 (13.3)	61,1 (7.8)

Rya åsar	Cleared	62.0 (8.6)	2.6 (0.8)

Rya åsar	Dense understory	81.0 (15.7)	113.2 (15.7)

Mean number of stems per 100 m^2 ^(S.E.) in relation to management at the two sites. 10 plots were sampled at each site.

### Experimental procedure

**Figure 4:**
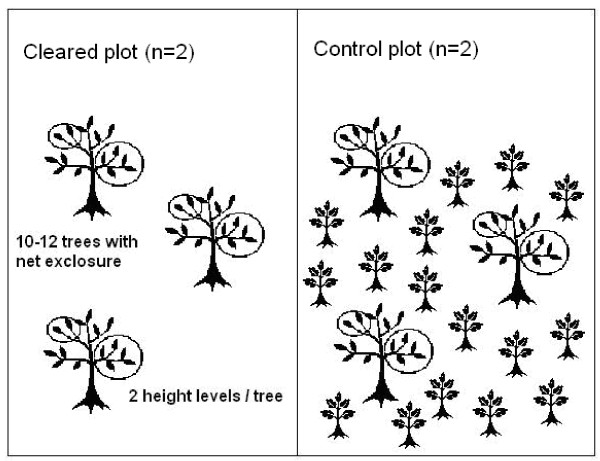
**Experimental setup**. The experiment was replicated at two sites. Each site was divided in two plots of equal area which were randomly assigned to either understory clearance or no management (control). Bird exclosures were placed in 10-12 trees in each plot, at two height levels in each tree.

The bird exclosure experiment lasted four weeks, starting 26-29 May 2008 and terminated 24-25 June. The experiment included 44 randomly selected oak trees (Figure 4), of which 24 were located at the Hultaberg site (12 trees in the cleared area and 12 trees in the control area) and 20 trees at the Rya åsar site (10 trees in the cleared area and 10 trees in the control area). Four branches were randomly selected at two different heights in each tree: two low branches (3-5 m above the ground) and two high branches (12-15 m above the ground). At each height one branch was enclosed with plastic anti-bird net (blue colour, mesh size 25 mm) to protect it from bird predation and one control branch was shaken to obtain a similar disturbance as for the net-enclosed branch. A portable lift with four-wheel drive and a 15 meter telescopic boom with a working platform was used to reach the branches. The trees were randomly selected from a map with a grid and a table of random numbers, although trees far away from paths or in wet or hilly terrain were discarded and new trees selected, as access with the lift was restricted to paths or relatively dry and flat terrain. The direction of branches at each height level was randomly selected using a compass and a table of random numbers.

### Collection of arthropods

Net-enclosed and control branches were cut from the trees as the experiment was terminated. Each branch was carefully enclosed in a large plastic sack before cutting. The sacks were then immediately sealed and stored at +4°C until examination in the laboratory. Branches were cut into smaller pieces (20-30 cm), which were examined over a large white tray. Arthropods were collected by hand and preserved in 70% ethanol. Arthropods with a body length less than 1 mm and aphids were not appropriately sampled by this method and were, therefore, not included in the analyses. A few, fast-flying insects escaped during the examination process, but notes were taken of the approximate size and order of these individuals. Immediately after arthropod collection, all leaves were removed from the branches and weighed. To control for differences in branch size, arthropod abundance and biomass were related to the wet leaf-mass of each branch, which was assumed to be proportional to the leaf area. All branches were collected during dry weather conditions to minimize the variation in leaf moisture content. The collected arthropods were identified to order and their length was measured to the nearest mm using a stereo microscope with a measuring ocular. Length was measured from the top of the head to the end of the abdomen (excluding antennae, spinnerets, etc.). Fresh body mass of each specimen was estimated from its length by using order-specific length-weight regression equations from Hodar [48].

### Statistical analysis

Arthropod abundance and biomass were calculated for each branch and related to leaf mass. In the analyses, we examined the effects of bird predation on the entire arthropod population (except Aphids and specimens <1 mm) as we wanted to avoid problems of mass-significance when performing multiple tests and for some orders the sampling variance was high (see Additional file 1), due to clumped distribution. The overall effect of net-exclosures was tested by pairwise comparisons of arthropod abundance and biomass, respectively, between paired branches with and without bird exclosure (paired samples t-test, 2-tailed). This test was carried out across sites, management types and height levels.

As the overall exclosure effect was found to be strong (see Results), we used the "Predation effect" as a measure of the avian predation rate, similarly to Van Bael *et al*. [49]. The "Predation effect", *E*, was calculated for each pair of branches (net-enclosed and control), as the relative increase in arthropod abundance or biomass on the net-enclosed branch relative to the control branch:(1)

where *N *is the abundance/biomass value of the net-enclosed branch and *C *is the value of the control branch. This measure will estimate the relative intensity of predation on canopy-living arthropods at two height levels (i.e. two values of *E *per tree). In this way we took advantage of the matched design within each tree and increased the possibility of detecting any difference in predation rate. A value of *E *indicates the strength of predation pressure and shows the factor by which *C *is to be multiplied to obtain the difference in abundance between *N *and *C*. (For instance, if *N *= 50 and *C *= 10 then *E *= 4 but if the difference is small, say *N *= 50 and *C *= 40 then *E *= 0.25). We assumed that abundances on branches at each height were similar at the start of the experiment (supported by earlier results [16,50]. Each pair of branches was treated as an independent sample. To test the independence, we conducted correlation tests (Spearman rank correlation) of "Predation effect", *E*, within trees. Separate tests were conducted for *E *on arthropod abundance and biomass.

Univariate analysis of variance (ANOVA) was used to test for Management (dense understory "control" vs cleared understory), Height (low vs high) and Site (Hultaberg, Rya åsar) effects, with the "Predation effect" as the response variable. Separate analyses were performed for "Predation effect" on arthropod abundance and biomass. The analyses were carried out using the GLM function in SPSS 12.0.1 for Windows with the model "Predation effect = Management + Height + Site + Management × Height + Management × Site + Height × Site". The second-order interaction (Management × Height × Site) was not included in the model as it was not a part of our hypothesis and moreover it was difficult to interpret. Management and Height were considered fixed factors while Site was treated as a random factor. Levene's test was used to test for heterogeneity of variances within samples. Predation effect data for arthropod biomass did not meet the assumption of homogeneity of variances and was therefore transformed (log (x+1)), which removed the heterogeneity [51]. All statistical analyses were performed using the SPSS 12.0.1 for Windows software.

## Authors' contributions

BG and EH contributed equally in the design of the study. EH carried out the field work with assistance of BG. The authors analyzed field data and drafted the manuscript together. Both authors read and approved the final manuscript.

## Supplementary Material

Additional file 1 **Abundance (numbers/kg wet leaf mass ± s.d.) and biomass (g/kg wet leaf mass ± s.d.) of separate arthropod orders (orders with >50 recorded individuals in total) in relation to management and experimental treatment**. The arthropod orders with <50 recorded individuals were: Lepidoptera, Opiliones, Neuroptera, Dermaptera, Acarina, Collembola, Ephemeroptera and Trichoptera. Click here for file
