# Supplementary material for: Management effect on bird and arthropod interaction in suburban woodlands
Source: BMC Ecol. 2011 Mar 1;11:8. doi: 10.1186/1472-6785-11-8 (PMC3058007; doi:10.1186/1472-6785-11-8)
Supplement: Additional file 1 — Abundance (numbers/kg wet leaf mass ± s.d.) and biomass (g/kg wet leaf mass ± s.d.) of separate arthropod orders (orders with >50 recorded individuals in total) in relation to management and experimental treatment. The arthropod orders with <50 recorded individuals were: Lepidoptera, Opiliones, Neuroptera, Dermaptera, Acarina, Collembola, Ephemeroptera and Trichoptera. [file 1472-6785-11-8-S1.DOC]

| **Abundance (numbers/kg wet leaf mass ± s.d.)** | | | | | | | |  |  |  |  |  |  |  |  |  |
| --- | --- | --- | --- | --- | --- | --- | --- | --- | --- | --- | --- | --- | --- | --- | --- | --- |
|  |  |  |  |  |  |  |  |  |  |  |  |  |  |  |  |  |
| **Management** | Dense understory |  |  |  |  |  |  |  | Cleared |  |  |  |  |  |  |  |
| **Treatment** | Control |  |  |  | Net |  |  |  | Control |  |  |  | Net |  |  | Total number |
| *Heteroptera* | 44,3 | ± | 38,9 |  | 68,9 | ± | 50,7 |  | 95,7 | ± | 82,5 |  | 101,6 | ± | 53,1 | 1372 |
| *Psocoptera* | 56,2 | ± | 51,2 |  | 48,2 | ± | 37,9 |  | 60,5 | ± | 70,7 |  | 56,9 | ± | 58,6 | 792 |
| *Aranae* | 44,3 | ± | 31,4 |  | 48,2 | ± | 28,2 |  | 47,4 | ± | 30,8 |  | 39,0 | ± | 25,0 | 746 |
| *Homoptera* | 31,8 | ± | 28,9 |  | 36,2 | ± | 31,1 |  | 34,5 | ± | 31,9 |  | 46,2 | ± | 38,6 | 717 |
| *Coleoptera* | 8,5 | ± | 10,3 |  | 24,7 | ± | 31,0 |  | 10,8 | ± | 14,6 |  | 26,8 | ± | 33,3 | 263 |
| *Hymenoptera* | 8,2 | ± | 12,3 |  | 7,3 | ± | 9,3 |  | 13,8 | ± | 17,2 |  | 14,7 | ± | 19,0 | 184 |
| *Lepidoptera (larvae)* | 5,8 | ± | 11,3 |  | 9,7 | ± | 13,8 |  | 5,1 | ± | 7,8 |  | 10,8 | ± | 11,9 | 137 |
| *Coleoptera (larvae)* | 4,3 | ± | 11,2 |  | 5,1 | ± | 9,2 |  | 6,6 | ± | 20,0 |  | 4,5 | ± | 9,7 | 59 |
| *Diptera* | 2,1 | ± | 5,0 |  | 7,4 | ± | 11,8 |  | 1,2 | ± | 4,5 |  | 4,2 | ± | 7,4 | 59 |
| *Neuroptera (larvae)* | 1,6 | ± | 4,5 |  | 2,9 | ± | 7,0 |  | 5,0 | ± | 10,6 |  | 6,5 | ± | 14,3 | 55 |
|  |  |  |  |  |  |  |  |  |  |  |  |  |  |  |  |  |
|  |  |  |  |  |  |  |  |  |  |  |  |  |  |  |  |  |
| **Biomass (wet arthropod mass/wet leaf mass g/kg ± s.d.)** | | | | | | | |  |  |  |  |  |  |  |  |  |
|  |  |  |  |  |  |  |  |  |  |  |  |  |  |  |  |  |
| **Management** | Dense understory |  |  |  |  |  |  |  | Cleared |  |  |  |  |  |  |  |
| **Treatment** | Control |  |  |  | Net |  |  |  | Control |  |  |  | Net |  |  | Total biomass |
| *Heteroptera* | 100,4 | ± | 124,4 |  | 293,8 | ± | 284,0 |  | 283,8 | ± | 365,1 |  | 401,1 | ± | 406,4 | 4920,7 |
| *Homoptera* | 82,4 | ± | 88,4 |  | 105,1 | ± | 112,9 |  | 65,0 | ± | 61,9 |  | 100,9 | ± | 82,4 | 1554,6 |
| *Lepidoptera (larvae)* | 39,7 | ± | 123,3 |  | 71,7 | ± | 127,2 |  | 22,8 | ± | 55,5 |  | 83,5 | ± | 158,6 | 1202,8 |
| *Coleoptera* | 36,2 | ± | 63,6 |  | 71,6 | ± | 104,8 |  | 38,4 | ± | 76,0 |  | 119,1 | ± | 169,4 | 1080,6 |
| *Aranae* | 31,1 | ± | 25,8 |  | 71,0 | ± | 55,1 |  | 40,7 | ± | 40,5 |  | 53,8 | ± | 43,5 | 956,8 |
| *Hymenoptera* | 6,9 | ± | 12,1 |  | 11,7 | ± | 36,1 |  | 8,7 | ± | 11,7 |  | 35,0 | ± | 100,5 | 250,7 |
| *Psocoptera* | 13,9 | ± | 13,3 |  | 12,6 | ± | 10,0 |  | 17,4 | ± | 23,2 |  | 15,8 | ± | 18,1 | 207,4 |
| *Neuroptera (larvae)* | 2,7 | ± | 9,5 |  | 8,6 | ± | 22,3 |  | 7,7 | ± | 17,9 |  | 23,4 | ± | 54,5 | 164,4 |
| *Diptera* | 2,0 | ± | 6,0 |  | 4,0 | ± | 8,4 |  | 1,7 | ± | 7,4 |  | 12,2 | ± | 41,9 | 85,9 |
| *Coleoptera (larvae)* | 4,6 | ± | 14,8 |  | 4,9 | ± | 10,3 |  | 4,0 | ± | 11,8 |  | 5,3 | ± | 13,4 | 53,5 |
